# Knowledge and understanding risk factors and preventive measures for osteoporosis in women: results of a survey in 502 women with and without a migration background

**DOI:** 10.1186/s12891-022-05773-0

**Published:** 2022-08-30

**Authors:** Reza Taghvaei, Desislava Dimitrova, Murat Karaman, Jalid Sehouli

**Affiliations:** 1 Department of Gynecology with Center for Oncological Surgery, Charité – Universitätsmedizin Berlin, corporate member of Freie Universität Berlin and Humboldt-Universität zu Berlin, Campus Virchow-Klinikum, Augustenburger Platz 1, 13353 Berlin, Germany; 2 Orthopedic Praxis Center Berlin, Berlin, Germany; 3 Statistical Analysis Agency, Berlin, Germany

**Keywords:** Migrants, Osteoporosis, Knowledge, Preventive measures

## Abstract

**Background:**

Osteoporosis is a disease of the skeletal system associated with loss of bone mass and an increased risk of fractures affecting women more often than men. Identification of the knowledge about osteoporosis and its preventive methods is the backbone of any awareness program. This study investigates the knowledge with a special focus on women with and without a migration background.

**Methods:**

Data from systematic patient interviews based on a questionnaire were collected at three different sites in Berlin between February and June 2021. The survey included questions assessing migrant background, demographic characteristics, lifestyle habits including physical exercise and smoking, prevention by vitamin D intake and bone densitometry, and information on personal and family medical history. According to the responses, a scale was created to assess the level of knowledge of preventive osteoporosis measures. The ethic committee of the Charité, Medical faculty has approved this study. SPSS (version 24.0) was used for the statistical analyses.

**Results:**

The survey of 502 female patients revealed that 25% had low and 34% no previous knowledge of osteoporosis. Older age and a better education level correlate with a higher knowledge. Patients with gynecologic cancer are less well informed. There is a significant difference in vitamin D intake between migrant and non-migrant women (57% vs. 49%). There were no significant differences regarding the use of bone densitometry.

**Conclusion:**

Knowledge of osteoporosis and the possibility of a bone densitometry as well as the implementation of preventive measures is low among women. Therefore, informing patients better should be a priority, with particular attention on the risks and needs of women with a migration background. Specific programs for women with and without migration background should be developed to increase the awareness of osteoporosis.

## Introduction

Osteoporosis is a disease of the skeletal system associated with a loss of bone mass and an increased risk of fractures. The lifetime risk of fracture is higher in women than in men [1]. An evaluation from 2006 to 2009 showed a fracture rate of 27% in those > 50 years of age [2]. More recent analyses show fracture rates of up to 30% in those > 70 years old and untreated for osteoporosis. This leads to a significant impact on patients’ quality of life and high costs for the health care system [3]. With increasing life expectancy, the number of elderly individuals is rising and the incidence of worldwide burden of fragility fractures is estimated to rise [4, 5]. Strategies to reduce this burden are needed by identifying individuals at risk and by increasing awareness of osteoporosis. The FRAX algorithm for example is a model, which enables the estimation of the patients’ absolute risk of fracture over the next 10 years [6].

Another important strategy as mentioned above is to increase patient awareness of osteoporosis risk factors by increasing their knowledge to encourage them to take preventive measures and to improve compliance with therapy [7].

Lifestyle factors such as smoking, high alcohol consumption, or lack of exercise also play a significant role in bone quality [8, 9]. Positive family history, vitamin D deficiency, reduced calcium intake, underweight (BMI < 20), and age are discussed as additional risks for increased fracture rates [10–13]. There is clear evidence such as increasing physical activity, cessation of smoking, adequate intake of vitamin D and calcium may reduce the risk of osteoporosis [14].

Awareness of such risks and the options for primary prevention is essential in reducing the incidence of osteoporosis. Several studies [15–17] have examined the association between knowledge of osteoporosis and preventive behavior. Some of them have shown that the level of knowledge could improve the use of preventive measures such as increasing exercise and calcium intake [18]. In addition, there are only few studies, that have used a validated tool to measure the knowledge of osteoporosis such as the following: Osteoporosis Knowledge Assessment Tool (OKAT) used for a survey in the Australian population [19]; the Osteoporosis Assessment Questionnaire, or OPAQ for postmenopausal women with established osteoporosis [20].

Based on our information, no similar studies have researched the level of knowledge of osteoporosis within migrants and non-migrants.

Specific frail groups in society, such as women with a migrant background seem to be confronted with significantly more impediments for taking up preventive measures and should therefore be specially considered [21]. Language barriers, poor working conditions, and low social status could increase the health risks for many migrant women [22, 23]. Increasing migration due to crisis situations in Europe and worldwide has led to ethnical and socio-cultural diversity in society and in health systems. These cohorts of patients should be reflected in clinical research [24]. Therefore, this survey aims to examine how women with and without a migrant background are informed and educated about bone health and the subsequent consequences of osteoporosis, including the sources of information most frequently used. The secondary aim is to investigate whether there are differences between migrant and non-migrant women regarding the use of preventive measures such as vitamin D intake and undergoing bone densitometry. We hypothesized the following: migration status could influence the level of knowledge and that migrants use preventive measures less often.

The results of this investigation could form a basis to develop new ways of informing patients and informational concepts.

## Methodology

The patient survey data were collected in the following three locations in Berlin, Germany from March to June 2021: the Department of Gynecology with Center for Oncological Surgery Charité Campus Virchow Klinikum, Orthopedic Praxis Center Berlin, and at the Gynecologic Praxis Berlin Mitte.

A power analysis was conducted to estimate the sample size for demonstrating a difference between two groups: migrants and non-migrants. A sample size of 210 participants was required to detect with an effect size of 0.5, power = 0.95 size (alpha = 0.05, two-tailed test).

A total of 502 women were enrolled: 202 at the Department of Gynecology, Charité Virchow-Klinikum; 150 at Orthopedic Center Berlin; and 150 at Gynpraxis Berlin Mitte (Table 1). Charité’s Berlin Ethics Committee approved the study (Reference number of ethics approval: EA4 / 02/21).

**Table 1:** Study population divided into 3 locations where the patients were recruited

		Orthopedic Praxis (*n* = 150)	Gynecologic Praxis (*n* = 150)	Virchow Clinic (*n* = 202)	*p*-value
BMI	< 20	8.7%	18.0%	17.30%	*p* ≤ 0,001
	20-30	53.3%	66.7%	65.8%	
	> 30	21.3%	14.0%	10.4%	
	> 35	17.7%	1.3%	6.4%	
School-leaving qualification	No qualification	10.2%	1.4%	2.6%	*p* ≤ 0,001
	High school	26.5%	2.8%	12.0%	
	Certificate of secondary education	26.5%	22.7%	27.6%	
	A-levels (UK) / SAT (USA)	14.3%	20.6%	17.7,%	
	University degree	22.4%	52.5%	40.1%	
Employment status	Employed	46.0%	75.3%	47.5%	*p* ≤ 0,001
	Unemployed	26.7%	16.0%	19.3%	
	Pensioner	27.3%	8.7%	33.2%	
Migration background	Yes	38.0%	36.7%	28.2%	*p* = 0,103
	No	62.0%	63.3%	71.8%	
German language skills	Very good	38.3%	49.1%	45.6%	*p* = 0,004
	Good	43.3%	10.5%	29.8%	
	Moderate	8.3%	14.0%	10.5%	
	Basic	10.0%	24.6%	8.8%	
	No skills	0.0%	1.8%	5.3%	
Pre-existing disease	Yes	66.0%	45.3%	58.4%	*p* = 0,001

The patients were given a patient information form and a questionnaire in German. Those aged 18 and over who could read the patient information and gave their informed consent to participate were included in the study. According to the ethics protocol, patient signatures were not required. This was to preserve anonymity on the informed consent form. The patient information form declared that by answering the questions, the respondent agreed to participate in this survey voluntarily, and the answers would be further analyzed and published. The questionnaire was answered anonymously. Therefore, no personal data that could directly identify a patient were gathered.

All the eligible female patients who visited the three locations for medical treatment and had adequate German language skills were included consecutively and were approached by trained medical staff. Male patients and females under 18 years were not included. When women with a migration background had poor German skills, medical staff with the relevant foreign language skills was called in to translate. Those who did not speak German were not included in the study when a translator in the required language was unavailable.

The survey consisted of 49 questions and was divided into three sections.

The patients were asked about their origin, language, religion, and education level in the first part.

The second section contained questions on general and gynecologic pre-existing diseases, lifestyle, physical activity, smoking, diagnostics, and treatments that had already been carried out. Finally, the third included assessing preventive measures such as vitamin D intake and knowledge of diagnostics about osteoporosis. The research questions were developed based on an interprofessional workshop between gynecologists, orthopedics and nurses and an intensive literature research. The aim of this survey was to collect data and generate a hypothesis as a basis for discussion and future strategies in patient care; not to develop and implement a new questionnaire. The comprehensibility was tested in a small group of 10 participants during a pilot phase. Therefore, no specific validation tests have been applied.

We performed an inferential analysis to generate and test the hypothesis that migrant background may influence the willingness to take part in preventive measures such as undergoing bone densitometry and vitamin D intake. We performed a descriptive analysis to distinguish factors that influence the population’s level of knowledge about osteoporosis. We conducted different subgroup analyses comparing migrants from the first and second generations and patients with or without gynecologic cancer. For the purpose of our study we defined that a person has a migration background if they or at least one parent was not born with German citizenship, as suggested from Federal Statistical Office in Germany [25, 26]. Migrants born abroad are considered first generation and those born in Germany second-generation.

In our study, the level of knowledge about preventive osteoporosis measures was calculated using a scale. After evaluating four questions from the questionnaire and their various positive and negative answer options, a 4-point scale was formed. The more positive answers the patients had, the more informed they were.

The four questions are as follows:

1. Have you ever had blood drawn for vitamin D tests?
2. Who has prescribed or recommended you vitamin D?
3. Has your family doctor ever approached you about bone health?
4. Have you ever had a bone densitometry in your life?

The evaluation was carried out according to the following scheme:

The patient received one point in the survey for each positively answered question. If patients received more points when answering, it was assumed that they had a higher level of knowledge regarding bone health. Therefore, according to the number of positive answers, the patients were divided into 4 categories where 0 represented absent; 1, low; 2, moderate; and 3, a high level of knowledge.

The data were evaluated using SPSS (version 24.0), a statistical data processing program. In addition to calculating the simple statistical values (absolute and relative frequencies), the Chi-Square Test was used to compare the relationship between two features. Furthermore, a binary logistic regression analysis was applied to independently check various influencing factors on vitamin D intake and on the probability of undergoing bone densitometry as a preventive measure. Finally, the impact of independent variables on the level of knowledge was evaluated using multivariate linear regression.

## Results

### Demographic data

A total of 502 patients were interviewed at the three locations, of which 65.3% (329) were non-migrants and 34.5% (173) were migrants. The latter represented first 26.1% (131) and 8.4% (42) second generation migrants. The proportion of migrants at the different locations was comparable: 36.7% at Gynpraxis Berlin Mitte, 38.0% at the Orthopedic Praxis Center Berlin, and 28.2% at the Department of Gynecology with Center for Oncological Surgery Charité Campus Virchow Klinikum (*p* = 0.103). The demographic characteristics of the patients from the three locations are summarized in Table 1.

The average age was 46 for migrants and 53 for non-migrants (range 22-84). Both groups’ average body mass index was 25 kg/m^2^ (range 14.4 - 50.8 kg/m^2^). The number of high-school and university graduates was higher among migrants: 57% vs. 51%.

Non-migrant women are employed more frequently than migrant women (59% vs. 48% *p* = 0.00). When asked about religious affiliations, 48% of non-migrants vs. 18% of migrants said they had no religion. In 30.8% of migrant women, they stated Christianity as their religion, and 40.2% Islam. In contrast, only 1.8% of non-migrant women declared Islam as their religion, and 37.8% Christianity (Table 2).

**Table 2:** Demographic data of the study population

*N* = 502		Migrants = 173	Non-migrants = 329	*p*-value
Average age		46.1 ± 14,3	52.7 ± 14,6	*p* ≤ 0,001
BMI		25.8 ± 6,2	25.5 ± 5,8	*p* = 0,452
Education / Employment status				
No school qualifications		8.9% (*n* = 15)	2.1% (*n* = 7)	*p* = 0,003
High school diploma / university degree		57.4% (*n* = 97)	51.3% (*n* = 171)	
Employed		47.9% (*n* = 81)	59.2% (*n* = 197)	*p* ≤ 0,001
Unemployed		33.7% (*n* = 57)	13.8% (*n* = 46)	
Retired		18.3% (*n* = 31)	27% (*n* = 90)	
Religion				
Christian		30.8% (*n* = 52)	37.8% (*n* = 126)	*p* ≤ 0,001
Islam		40.2% (*n* = 68)	1.8% (*n* = 6)	
Other religion		10.7% (*n* = 18))	12.70% (*n* = 42)	
No religion		18.3% (*n* = 31)	47.7% (*n* = 159)	
Pre-existing diseases				
Pre-existing disease yes		50.9% (*n* = 86)	59.8% (*n* = 199)	*p* = 0,058
Pre-existing disease no		49.1% (*n* = 83)	40.2% (*n* = 134)	
Obesity (BMI > 35)		36.0% (*n* = 18)	64.0% (*n* = 32)	*p* = 0,713
Underweight (BMI < 18)		36.8% (*n* = 7)	63.2% (*n* = 12)	*p* = 0,765
Cardiovascular disease		24.0% (*n* = 18)	76.0% (*n* = 57)	*p* = 0,055
Diabetes		33.3% (*n* = 11)	66.7% (*n* = 22)	*p* = 0,976
Thyroid disease		31.1% (*n* = 38)	68.9% (*n* = 84)	*p* = 0,499
Rheumatic diesease		38.5% (*n* = 10)	61.5% (*n* = 16)	*p* = 0,800
Malignant gynecologic cancer		22.40% (*n* = 37)	30.1% (*n* = 98)	*p* = 0,073
Physical Exercise	Yes	59% (*n* = 102)	65% (*n* = 214)	*p* = 0,391
	No	41% (*n* = 67)		
Smoking	Yes	15.6% (*n* = 27)	21% (*n* = 69)	*p* = 0,007
	No	84.4% (*n* = 142)		
Alcohol consumption	Yes	34.7% (*n* = 60)	54.4% (*n* = 179)	*p* ≤ 0,001
	No	65.3% (*n* = 109)	45.6% (*n* = 154)	

Regarding the frequency and type of previous illnesses relevant to bone health, there are apparent differences between migrants and non-migrants. Non-migrants had secondary diseases (*p* = 0.05) more frequently such as obesity, underweight, cardiovascular diseases, diabetes, thyroid diseases, rheumatic disease, and gynecologic tumor diseases compared to migrant women (Table 2).

A positive family history of osteoporosis in the two groups (migrants and non-migrants) was indicated as 21%. In their personal medical history, 25% of the patients had suffered a fracture without trauma, and 18.5%, had had a fall.

### Lifestyle factors

When analyzing the lifestyle factors, it is noticeable that migrant women exercise less on a daily basis (59% vs. 65% - *p* = 0.39), smoke less (15.6% vs. 21% - *p* = 0.007) and consume significantly less alcohol (34.7% vs. 54.4% - *p* = 0.000) (Table 2).

### Preventive measures and sources of information

Most of the patients answered that the orthopedist (72%) was the doctor responsible for their bone health. However, the family doctor was the one who recommended taking vitamin D as prophylaxis for osteoporosis (27.5%), though they rarely requested further diagnostics (9.2%). In their assessment of bone health, 76.9% rated their bone quality as good.

There are no significant differences between migrant women and those without a migrant background in the abovementioned analyses.

There was a significant difference in vitamin D intake between migrant and non-migrant women (57% vs. 49%, *p* = 0.041). The multivariate analysis of vitamin D intake and possible influencing factors in the total population shows the following result: a higher level of education (*p* = 0.000), a migration background (*p* = 0.009), suffering from a rheumatic disease (*p* = 0.020) or ovarian cancer (*p* = 0.029), regular physical activity (*p* = 0.032), and the use of medication (*p* = 0.037) are direct factors that influence the intake of vitamin D (Table 3).

**Table 3:** Multivariate analysis on influencing factors on vitamin D intake in the overall population and among migrant and non-migrant women

Vitamin intake D Total Population	*p*-value	OR^a^	Vitamin D intake		*p*-value	OR
School-leaving qualification	0.000	1.346	Migrants	School-leaving qualification	0.554	1.099
Migrant background	0.009	1.721		Rheumatism	0.223	0.37
Rheumatic disease	0.020	3.231		Medication	0.705	0.814
Ovarian cancer	0.029	1.942	Non-migrants	School-leaving qualification	0.000	2.075
Exercise	0.032	1.569		Rheumatic disease	0.002	29.322
Medication	0.037	1.557		Medication	0.037	0.413

^a^*OR* Odds Ratio

The factors influencing the intake of vitamin D for non-migrant women are as follows when comparing migrants and non-migrants: level of education, exercise, and medication. Furthermore, in the subgroup analysis for migrant women, no significant influencing factors could be shown (Table 3).

Twenty-one percent of women were aware of early diagnosis by undergoing a bone densitometry as part of preventive care for women over 60 years of age; 24% had already undergone an examination. There were no significant differences between migrant and non-migrant women in the use of a bone densitometry.

Regarding the diagnosis of osteoporosis using bone densitometry in the total population, the multivariate analysis shows a significant result for the influencing factors age (*p* = 0.000), physical exercise (*p* = 0.003), and positive family history (*p* = 0.035) (Table 4).

**Table 4:** Multivariate analysis on influencing factors associated with bone densitometry in the past in the overall population and in migrant and non-migrant women

	*p*-value	OR^a^	Bone densitometry		*p*-value	OR
Age	0.000	0.906	Migrants	Age	0.000	0.899
BMI	0.109	0.962		Rheumatic disease	0.432	2.325
Migration background	0.240	0.707		Underweight	0.024	0.060
Rheumatic disease	0.109	0.402		Exercise	0.773	1.205
Diabetes	0.102	0.46	Non-migrants	Age	0.000	0.912
Ovarian cancer	0.109	0.583		BMI	0.517	0.979
Excercise	0.003	0.417		Rheumatic disease	0.043	0.213
Positive family history (Osteoporosis)	0.035	0.53		Underweight	0.505	1.926
				Exercise	0.023	2.464

^a^*OR* Odds Ratio

In addition to age (*p* = 0.000) and exercise (*p* = 0.023), the presence of rheumatic disease (*p* = 0.043) is an important factor within non-migrant women. In migrant women, age (*p* = 0.000) and underweight (*p* = 0.024) have an influence on the use of the bone densitometry (Table 4).

The subgroup analyses were divided into first and second generations to evaluate vitamin D intake and bone densitometry within migrant women. There was no significant difference between first- and second-generation migrants in terms of vitamin D intake and bone densitometry (data not shown).

We analysed the influence of the number of years of residence in Germany and did not find any significant influence of the results. Therefore, the data were not further included in the multivariate regression analysis.

When examining only patients with gynecologic cancer, the following influencing factors for vitamin D intake and for performing bone densitometry were confirmed: BMI (*p* = 0.030), physical exercise (*p* = 0.023), and medication intake (*p* = 0.010) (Table 5).

**Table 5:** Mutivariate analysis on influencing factors for vitamin D intake and performing a bone densitometry based on patient history of gynecologic cancer

		Vitamin D		Bone densitometry	
		*p*-value	OR	*p*-value	OR
Patients	BMI	0.030	1.556	0.193	0.928
with	Age	0.655	1.008	0.000	0.921
Gynecological	Excercise	0.023	0.023	0.087	0.405
Cancer	Medication	0.010	0.010	0.431	0.606
	Diabetes	0.945	1.075	0.350	0.387
	Rheumatic diesease	0.387	2.590	0.682	0.654
	Thyroid disorder	0.545	0.719	0.697	1.237
	Obesity	0.065	0.111	0.136	5.906
	Underweight	0.062	10.333	0.536	0.493
	Cardiovascular disease	0.295	0.524	0.605	1.366
	Smoking	0.116	0.321	0.933	1.062
	Alcohol consumption	0.398	0.644	0.323	1.684
	Positive family history	0.908	1.058	0.311	0.609
Patients	BMI	0.300	0.970	0.402	0.964
without	Age	0.932	1.001	0.000	0.903
Gynecolocical	Exercise	0.466	0.773	0.020	0.366
Cancer	Medication	0.465	1.244	0.096	0.457
	School-leaving qualification	0.073	1.235	0.119	1.307
	Diabetes	0.735	1.195	0.323	0.503
	Rheumatic diesease	0.024	4.127	0.236	0.384
	Obesity	0.658	1.264	0.513	0.627
	Underweight	0.491	1.857	0.187	0.218
	Cardiovascular disease	0.404	0.714	0.740	1.184
	Smoking	0.495	0.815	0.588	0.783
	Alcohol consumption	0.762	1.081	0.190	0.581
	Positive family history	0.359	1.319	0.021	0.352

The evaluation of the study population’s level of knowledge about osteoporosis and preventive measures showed that 161 (34%) of the respondents had no previous knowledge, 121 (25%) low, 148 (31%) moderate, and only 50 (10%) good previous knowledge.

In the multivariate analysis of these results, the following were found to be significant influencing factors: age (*p* = 0.001), level of education (*p* = 0.012), gynecologic cancer (*p* = 0.025), and underweight (*p* = 0.011) (Table 6). Migrant status did not significantly influence the level of knowledge.

**Table 6:** Multivariate linear regression analysis on factors influencing good level of knowledge

Factors	Standardized coefficients ßeta	t-value	*p*-value
Age	0.313	3.535	0.001
BMI	0.081	0.669	0.505
School-leaving qualification	0.234	2.545	0.012
German language skills	0.020	0.247	0.805
Migration background	−0.023	−0.291	0.772
Gynecologic cancer	−0.187	−2.266	0.025
Rheumatic diesase	−0.131	−1.691	0.093
Diabetes	0.083	0.958	0.340
Thyroid disorder	0.052	0.637	0.525
Obesity	−0.057	−0.531	0.597
Underweight	0.208	2.575	0.011
Cardiovascular disease	−0.011	−0.119	0.905

Age, education, and underweight correlate positively with the level of knowledge. The higher the age and level of education, the greater the patient’s knowledge was. The correlation with the presence of gynecologic cancer was negative. Patients with the mentioned disease were comparatively less informed.

## Discussion

The focus of our study is to assess the level knowledge about bone health and osteoporosis among migrants in comparison to non-migrants. Due to the current global situation and the increasing relevance of migration and refugees movements, there is an increasing need to study further ethnical and socio-cultural aspects of the society and to include minorities and fragile groups in health research.

The reason for the selective screening of women is that they represent a considerable risk group for osteoporosis and possible complications. By explicitly including patients with a migrant background we aimed to reflect the ethnical and cultural diversity of the population. The survey included a sample from the Berlin population. The percentage of migrants in Berlin is estimated to be 34.7%, which is much higher than in the rest of Germany, comparatively. The percentage of migrants in our study was 34.5%, exactly within the range of the Microcensus 2020 data. A Microcensus is an official annual survey of 1% of German households and is conducted by the Federal Statistical Office of Germany [27].

According to our study results, only 10% of the women surveyed had good and 31% moderate previous knowledge of bone health, vitamin D intake, and bone densitometry. Three groups of patients were better preventive measures: older patients (*p* = 0.001), patients with a higher educational level (*p* = 0.012), and underweight patients (*p* = 0.011) (Table 6). The migrant status did not significantly influence the level of knowledge.

In our study, the percentage of participants with a good level of knowledge of osteoporosis was 41% and was similar to the results of other international studies. The majority of our patients had low to no level of knowledge. In contrast, studies from Turkey, around 45% of the women interviewed knew the correct definition of osteoporosis [28, 29]. It was similar in Lebanon with 45% [30] and higher in Canada by 61% [31]. Data from Singapore showed that 54% of the women surveyed were aware of osteoporosis [32]. In a Saudi Arabian study, 59.8% knew about this illness well [33].

A study on the supply of vitamin D carried out in the German adult population showed that 61.6% of the participants had an insufficiency and 30.2% a deficiency of vitamin D [34].. People with darker skin pigmentation or those who wear a veil for cultural reasons are particularly susceptible to a vitamin D deficiency [35]. In addition, a study on vitamin supply in children and adolescents confirmed the association between vitamin D deficiency and migration background [36]. More than half of the migrant women in the present study population (57%) took vitamin D and migrants took it more often than non-migrants.

It can be assumed that migrant women are confronted with the issue of vitamin D deficiency at an early stage and therefore pay more attention to the prophylactic intake of this vitamin compared to non-migrants.

In rheumatoid arthritis, a vitamin D deficiency can be associated with diffuse musculoskeletal pain and the severity of the illness. Vitamin D has been used successfully for pain relief in this group of patients [37]. Therefore, this observation could hypothetically explain the association between the presence of rheumatoid arthritis and vitamin D intake. Rheumatoid arthritis is associated with increased risk of osteoporotic fractures and osteoporosis is found in a large percent among patients with rheumatoid arthritis [38, 39]. Therefore, we evaluated if a presence of rheumatic disease could influence the vitamin D intake of the participants and found a significant association.

Informing patients about osteoporosis and possible preventive measures are particularly important for maintaining bone health. In similar studies from Turkey, Canada, Singapore, and the United States, patients stated that the most important means of receiving information are television and their treating physicians [28, 29, 40, 41]. In our survey, 72% said that the orthopedist was their most important source. However, the recommendation for vitamin D intake was mostly made by the family doctor.

An increase in general physical activity is substantial as a preventive measure. Data from a study on the health status of adults in Germany shows that migrants do physical exercise less often than non-migrants [42, 43]. In our survey, migrant women also indicated doing less physical activity on a daily basis. Regarding other relevant lifestyle factors for osteoporosis, our study shows that migrant women smoke less and consume less alcohol. Data from the 2017 Microcensus confirms that first-generation migrant women smoke less than non-migrants and second-generation migrants [44]. In a survey carried out in Saudi Arabia, 67.8% of women knew that smoking was a risk factor for bone health [33]. Lebanese studies from 2018 show a significantly lower level of knowledge: only 36% of postmenopausal women considered smoking a risk factor, and only 15% in a study from Pakistan [45].

According to the 2015 German Socio-Economic Panel study (SOEP), the prevalence of cardiovascular diseases, diabetes, and chronic back problems is lower in women with a migration background [46]. Our survey results confirm that migrants had fewer pre-existing diseases associated with bone health compared to non-migrants.

Regarding the possibility of bone densitometry as part of preventive care, 21% of the respondents were aware, and 24% had already undergone one. Furthermore, there was no difference between migrants and non-migrants in performing bone densitometry.

The objective of our study was to assess the level of knowledge of osteoporosis in the study population and to investigate, if the participants follow specific procedures to prevent osteoporosis. Although we collected data concerning individual risk factors such as smoking, alcohol consumption, positive family history, previous fractures, relevant systemic diseases such as rheumatic diseases, age, height we did not use the FRAX (Fracture Risk Assessment Tool) algorithm [6], which assess the risk of suffering an osteoporosis-related fracture in the next 10 years. We believe the evaluation of individual risk is of great importance and should be addressed in future studies.

Our survey results show that women with gynecologic cancer in the study population were less informed. A study among postmenopausal breast cancer survivors showed low mean score concerning osteoporosis knowledge. Only 47% reported commitment in strength-training exercise [47]. Another study evaluating osteoporosis knowledge in women with premature ovarian insufficiency and early menopause showed that there are knowledge gaps regarding risk factors and treatment options [48]. Patients who suffer from premature menopause due to bilateral salpingo-oophorectomy as treatment for gynecologic cancer or genetic predisposition such as BRCA mutation have an increased risk of osteopenia and osteoporosis [49, 50]. Therefore, the current data illustrates the need for specific and intensive informing about osteoporosis risks and prevention among women with gynecologic cancer.

In our study, a higher level of education was associated with a higher level of knowledge of osteoporosis. A Turkish study on bone health also confirmed the influence of education on osteoporosis knowledge concerning bone health and preventive measures [28].

After dividing the population into migrant and non-migrant women, the multivariate analysis showed no significant factors influencing vitamin D in migrants. However, the following factors remained significant among non-migrants: higher education, exercise, and medication use.

In the multivariate analysis, age, physical activity, and positive family history were found to be important influencing factors for performing bone densitometry. Based on the study results, it can be assumed that patients who are familiar with osteoporosis due to family medical history as well as older ones use diagnostics more often or are at least better informed. A study from Saudi Arabia confirms a connection between higher age (above 40) and being better informed [33]. In Turkey, on the other hand, the younger and better educated the patients were, the more informed they were [28].

A survey conducted in New Zealand in 2007 with 622 female participants showed that 22% of the respondents were aware that having a positive family history of osteoporosis was relevant to their bone health [51].

The multivariate analysis according to the subdivision of migrants and non-migrants shows that age and underweight for the former and age, rheumatic disease, and exercise for the latter have a significant influence on the use of bone densitometry. Therefore, one possible explanation would be that patients who suffer from a relevant pre-existing condition, such as rheumatoid arthritis or underweight, are offered a bone densitometry more often as part of their check-ups.

There were no significant differences in diagnostics and preventive measures among first and second-generation migrant women regarding knowledge of bone health.

One limitation of our study is that it included a selected group of patients from three health institutions in Berlin. Future research should include a larger population from different regions of Germany. Another bias arose because the interviewees were only ones who visited a hospital or a medical practice. These women may be more interested in health related topics and are therefore better informed. It would be helpful to carry out a survey at places independent of the health care system. This survey was only conducted in German, so migrant women with limited German skills and illiterates were not included. Therefore, our findings cannot translate to these cohorts of women. These patients should be involved in future studies.

Despite all methodical limitations we believe that the results of the present study provide significant insights and underline the need for further studies and specific awareness campaign for women with migration background.

In summary, knowledge of osteoporosis and the option of bone densitometry as well as taking preventive measures is low among women. Hence, it is necessary to promote awareness in this area. The majority of the women surveyed were unaware of the risk factors and consequences. Therefore, better patient information and the development of prevention strategies should be the focus of attention. An individual risk assessment of women should be carried out. In particular, women with gynecologic cancer should be informed about risk factors and preventive measures of osteoporosis. Specific programs for women with and without migration background should be developed to increase the awareness of osteoporosis.

## Data Availability

The datasets used or analysed during the current study are available from the corresponding author upon request.
